# Excluding Lung Tissue from the PTV during Internal Mammary Irradiation. A Safe Technique for OAR-Sparing?

**DOI:** 10.3390/cancers13081951

**Published:** 2021-04-18

**Authors:** Kai J. Borm, Christopher Hofmann, Mathias Düsberg, Markus Oechsner, Hendrik Dapper, Michal Devecka, Stephanie E. Combs

**Affiliations:** 1 Department of Radiation Oncology, Klinikum rechts der Isar, Technical University of Munich (TUM), Ismaninger Straße 22, 81675 Munich, Germany; christopher.hofmann@tum.de (C.H.); mathias.duesberg@tum.de (M.D.); Markus.oechsner@tum.de (M.O.); Hendrik.dapper@tum.de (H.D.); Michal.devecka@tum.de (M.D.); 2 Deutsches Konsortium für Translationale Krebsforschung (DKTK), Partner Site Munich, 81675 Munich, Germany; 3 Institute of Radiation Medicine, Helmholtz Zentrum München, 85764 Munich, Germany

**Keywords:** breast cancer, radiotherapy, internal mammary irradiation, organ-sparing, IGRT

## Abstract

**Simple Summary:**

The planning treatment volume (PTV) during internal mammary irradiation (IMNI) regularly overlaps with lung tissue and is often in close proximity to the heart. Thus, exclusion of lung tissue from the PTV is a potential technique to spare the organs at risk (OARs) during adjuvant breast cancer irradiation. Using an innovative dose recalculation and accumulation algorithm, we evaluated the safety of exclusion of lung tissue from the PTV. According to our data, exclusion of lung tissue from the PTV to spare the OARs leads to significant dose reduction in the target volume and can, therefore, not be recommended.

**Abstract:**

The current study aims to determine whether exclusion of lung tissue from planning treatment volume (PTV) is a valid organ at risk (OAR)-sparing technique during internal mammary irradiation (IMNI). Twenty patients with left-sided breast cancer undergoing adjuvant radiotherapy including IMNI after mastectomy or lumpectomy with daily ConeBeam CT (CBCT; median n = 28) were enrolled in the current study. The daily dose distribution of the patients was estimated by recalculating treatment plans on CBCT-scans based on a standard PTV (PTV margin: 5mm-STD) and a modified PTV, which excluded overlapping lung tissue (ExLung). Using 3D-deformable dose accumulation, the dose coverage in the target volume was estimated in dependence of the PTV-margins. The estimated delivered dose in the IMN-CTV was significantly lower for the ExLung PTV compared to the STD PTV: ExLung: V95%: 76.6 ± 22.9%; V90%: 89.6 ± 13.2%, STD: V95%: 95.6 ± 7.4%; V90%: 99.1 ± 2.7%. Daily CBCT imaging cannot sufficiently compensate the anatomic changes and intrafraction movement throughout the treatment. Therefore, to ensure adequate delivery of the prescribed dose to the IMN-CTV, exclusion of lung tissue from the PTV to spare the OARs is not recommended.

## 1. Introduction

Nodal-positive high-risk breast cancer patients are treated with adjuvant regional lymph node irradiation (RNI) to eradicate microscopic tumor spread in the lymphatic drainage system. According to randomized trials, RNI lowers regional lymph node recurrences and distant metastases and improves disease specific survival and overall survival [1,2]. The most frequent location for lymph node metastases (LNM) is the axillary region [3]. Involvement of the axilla is evaluated by clinical examination, imaging and most importantly surgical assessment performing either sentinel node biopsy (SLN) or axillary node dissection (ALND). For the internal mammary lymph node (IMN) region, however, surgical assessment is not a standard procedure and the limited sensitivity of CT imaging and ultrasound impedes reliable diagnosis of IMN metastases [4,5].

Older studies performing surgical IMN evaluation reported consistently that medial tumors and positive axillary nodes are strongly associated with an increased rate of IMN involvement (44–65%). Even in lateral tumors with negative axillary nodes, IMN metastases occurred in 4–13% [6,7]. This is accordance with lymphoscintigraphy studies revealing primary drainage to the IMNs in 30% of medial tumors and 15% of lateral tumors [6]. Thus, microscopic (and macroscopic) tumor spread in the IMN needs to be expected in a relevant number of node-positive breast cancer patients.

Radiotherapy contributes to the eradication of tumor cells in the IMN region and plays therefore an important role in node-positive breast cancer patients: A recent meta-analysis of randomized RNI trials concludes that the survival benefit of RNI depends on the inclusion of the internal mammary region [8]. The importance of internal mammary region irradiation (IMNI) is further evident from trials with either prospective or retrospective study design revealing a significant OS benefit (3.7–4.8%, *p* < 0.05) or a trend towards a better OS (3.3%, *p* = 0.8) after IMNI [9,10,11]. Based on the available evidence, inclusion of the IMN during RNI is consistently recommended in national and international guidelines [12,13]. Due to the proximity of the internal mammary chain to the heart and lung, IMNI increases the doses to the organs at risk [14]. Since the dose distribution in the organ at risk (OARs) is clearly correlated to adverse events after RT, there are concerns regarding long term cardiac and pulmonary toxicity after IMNI [15,16]. So far, reliable clinical data for side effects after IMNI using modern treatment techniques are lacking. Modern treatment techniques for OAR-sparing during RNI comprise CT-based 3DCRT, volumetric arc therapy (VMAT) and deep inspiration breath hold (DIBH) [17].

Due to precise irradiation techniques, target volume definition in the CT-images is gaining importance. While several international consensus contouring-recommendations address the definition of the clinical target volume (CTV) during RNI [18,19], standardized definitions regarding the PTV margins are missing. Pattern of care surveys reveal, that most radiation oncologists use a CTV to PTV margin of 5 mm during RNI to compensate position and treatment inaccuracies [20]. However, up to 40% of radiation oncologists [20] exclude lung tissue from the PTV during RNI in order to reduce the dose to the OARs [21,22,23]. This decreases drastically the dorsal PTV-margin of the IMN region. So far, literature lacks data regarding this technique and the effect on IMNI dose coverage remains unclear.

Estimation of the effect of CTV to PTV margins on the delivered dose to the CTV is challenging. For the current study we developed an innovative dose accumulation workflow based on non-rigid image registration that allows investigation of the effect of exclusion of lung tissue from the PTV on the OAR dose and the dose coverage in the CTV. The aim of this study was to evaluate whether this approach can be safely applied during IMNI.

## 2. Materials and Methods

### 2.1. Patients

For the current analysis, 20 breast cancer patients were selected who were treated with adjuvant radiotherapy to the breast or chest wall with RNI (consistent of supra-/infraclavicular irradiation and IMNI). All patients underwent daily (20–28) cone-beam computed tomography (CBCT). The median height of the included patients was 168 cm (155–181 cm), the median weight 70 kg (47.5–98 kg) and the median BMI 26.4 kg/m^2^ (19.5–31.3 kg/m^2^). Eleven patients underwent lumpectomy and nine received a mastectomy.

### 2.2. Radiotherapy Planning

For all patients, a planning computed tomography dataset was acquired on a Somatom Emotion 16 scanner (Siemens Healthineers, Erlangen, Germany) in free breathing. For each dataset the OARs (left anterior descending artery (LAD), heart, lung, contralateral breast) as well as the clinical target volumes according to the European Society for Radi Onotherapy and Oncology (ESTRO) contouring guideline [19] were contoured. Retrospectively, two different planning target volumes were defined for each patient:

(1) a standard PTV with a 5 mm CTV-PTV safety margin around the lymph node areas including the IMN
(2) a modified PTV with a 5 mm CTV-PTV safety margin around the lymph node areas including the IMN excluding the overlapping lung volumes from the PTV

Based on these target volumes two treatment plans were created for each patient, retrospectively.

(1) a standard treatment plan (STD) based on the standard PTV
(2) an alternative treatment plan (ExLung) based on the PTV excluding the overlapping lung tissue

All treatment plans (n = 40) were created in Eclipse 15.6 (Varian Medical Systems, Palo Alto, CA, USA) treatment planning system (TPS) in VMAT. The prescribed dose for all patients was 50.4 Gy (single dose 1.8 Gy, 5 fractions/week). Overall, 95% of the PTV should receive 90% of the prescribed dose and 95% of the IMN CTV 95% of the prescribed dose [24]. Dose maximum should not exceed 110% and preferably not 107%. The dose to OARs was kept as low as possible, without compromising the PTV dose.

### 2.3. Dose Analyses

The dose volume histograms (DVHs) of the organs at risk (heart, lung, contralateral breast) in the planning CT scans were exported and analyzed using “R” (R Foundation for Statistical Computing, Wien, Austria). Based on the DVHs, the planned dose values in the organs at risk (Dmean, Dmax, V20) and the target volume (V95%, Dmin, Dmax, Dmean) were assessed.

**Figure 1:**
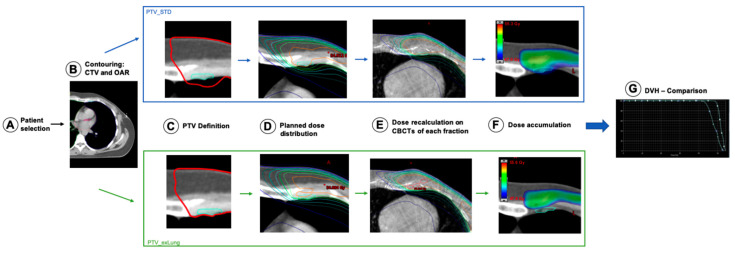
Methodology to determine differences of the dose distribution in the internal mammary lymph node- clinical target volume (IMN-CTV) in dependence of the planning safety margins during treatment. (**A**) Selection of patients meeting the selection criteria, (**B**) Contouring the IMN-CTV and the organs at risk, (**C**) Creation of two different PTVs using a 5 mm CTV-PTV margin–one including lung tissue (PTV_STD)–one excluding lung tissue (PTV_exLung), (**D**) Creation of the two corresponding irradiation plans and evaluation of “planned” dose distribution, (**E**) Dose recalculation based on each ConeBeam CT for each fraction (Color lines represent aquivalent isodoses), (**F**) Accumulation of the dose in each CBCT on the geometry of the planning CT, (**G**) Evaluation of the CBCT-based accumulated dose in the IMNI-CTV.

In a next step, a dose accumulation workflow was implemented to estimate the actual delivered dose over all fractions. For this, the dose distribution was recalculated on every CBCT acquired during the treatment of a patient (median: n = 28; min: n = 20 max: n = 28) in Eclipse. Non-rigid image registration was performed to assess a deformation vector field (DVF), projecting the CBCTs on the planning CT. The recalculated dose was deformed by the DVF and accumulated in a script, written in MATLAB 2019b (The MathWorks Inc., Natick, MA, USA) utilizing the image processing framework plastimatch. Figure 1 outlines the methodology graphically step-by-step.

For evaluation, the accumulated doses were reimported into the TPS. Dose coverage in the IMN-CTV was compared between ExLung and the STD treatment plan. Evaluation of the accumulated dose in the OARs was not performed, since the OARs were not completely depicted by the CBCT (due to the limited field of view). The workflow is delineated in Figure 1.

To estimate statistical significance of dose differences the Wilcoxon signed-rank test was used. This pairwise test is sensitive to the existence of plan differences but independent of the magnitude of this difference. *p*-values < 0.05 were considered statistically significant.

## 3. Results

### 3.1. OAR Dose

**Figure 2:**
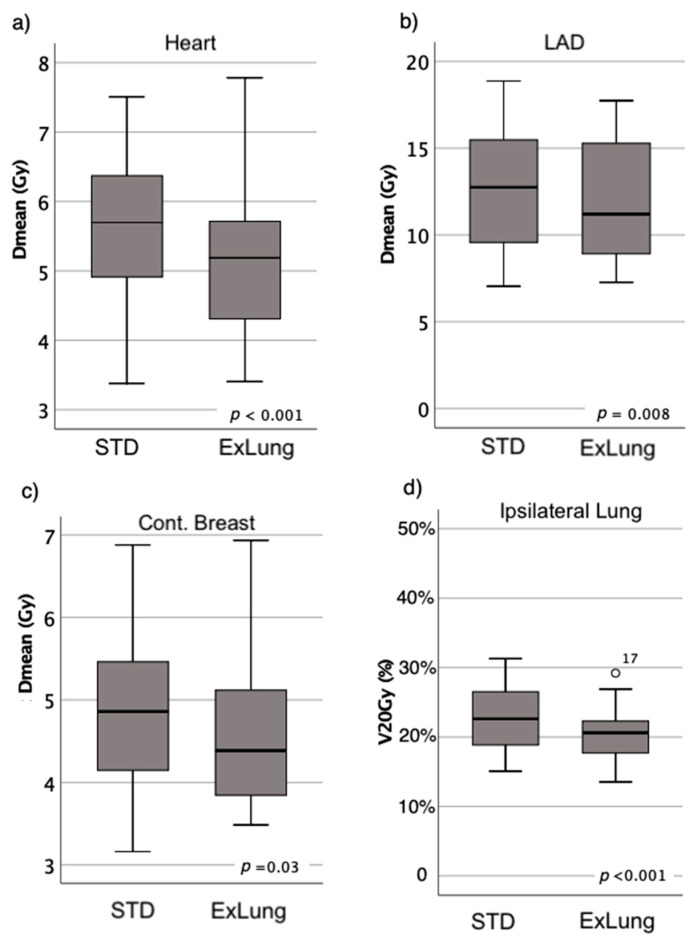
Boxplots of the dose distribution in the OARs in dependence of the safety margins. Standard treatment plan (STD): PTV = CTV(IMN)+ 5 mm; ExLung: PTV = CTV(IMN)+5 mm excluding lung tissue. Comparison of (**a**) Heart Dmean (**b**) Dmean in Left Anterior Descending Artery (LAD) (**c**) Contralateral Breast (**d**) Ipsilateral Lung.

The planned doses in the OARs in the treatment plans are summarized as box plot diagrams in Figure 2. Excluding lung tissue from the PTV during IMNI resulted in significant lower planned doses in the heart, LAD, ipsilateral lung and contralateral breast compared to the standard treatment plan with a 5 mm safety margin in all directions around the IMN-CTV. The median differences between the two techniques were 0.41 Gy for the mean heart dose, 0.82 Gy for the mean LAD dose, 1.2% for the V20Gy of the ipsilateral lung and 1.1 Gy for the contralateral breast.

### 3.2. IMN-CTV Coverage

The planned mean dose in the IMN-CTV, based on the planning CT, was 51.7 ± 1.1 Gy for the STD treatment plan and 51.4 ± 0.5 Gy for the ExLung treatment plan. The V95% was 99.8 ± 0.3% (STD) and 96.4 ± 2.7% (ExLung), the V90% 100 ± 0% (STD) and 99.8 ± 0.3% (ExLung).

**Figure 3:**
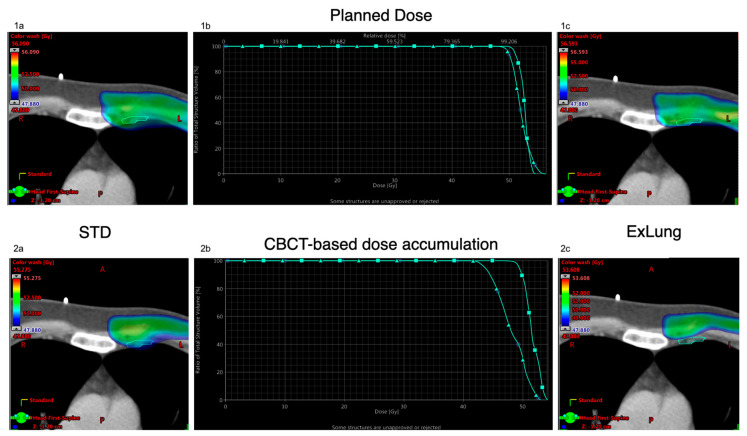
Impact of excluding lung tissue from the PTV. Delineation of the “planned” dose in the planning CT (**1a**–**c**) and the accumulated dose based on daily CBCT (**2a**–**c**) for the standard treatment plan using a 5 mm margin around the IMN-CTV (STD: **1a**,**2a**)) and for the a treatment plan with a 5 mm planning treatment volume (PTV) margin excluding overlapping lung tissue (ExLung: **1c**,**2c**).

**Figure 4:**
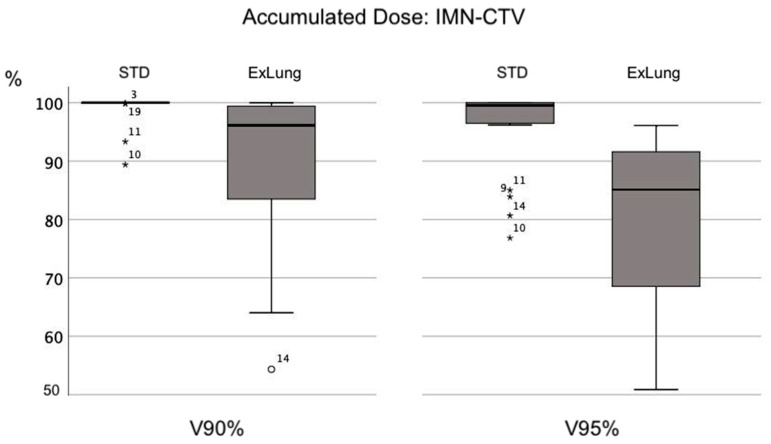
Box plot diagrams highlighting the impact of excluding lung tissue from the PTV on accumulated dose in the IMN-CTV. Outliers are marked with a circle. Extreme values are marked with an asterisk (*).

Recalculation and accumulation of the dose distribution in the CBCTs revealed reduction of the IMN-CTV dose coverage for both the STD treatment plan and for the ExLung compared to the planned dose based in the planning CT. While for the STD treatment plans, the CBCT dose accumulation lead still to an acceptable dose coverage in the IMN-CTV (mean: 51.4 ± 1.5 Gy; V95%: 95.6 ± 7.4%; V90%: 99.1 ± 2.7%), exclusion of lung tissue from the PTV (ExLung) resulted in a large variability of IMN-CTV dose coverage and significant lower average values (mean: 49.7 ± 1.9 Gy; V95%: 76.6 ± 22.9%; V90%: 89.6 ± 13.2%). Figure 3 delineates the dose distribution based in the planning CT and based on the daily CBCTs for the STD and the ExLung treatment plan in an exemplary patient. Figure 4 summarizes the dose coverage in the IMN-CTV based on CBCT recalculations for all 20 patients.

## 4. Discussion

Our results indicate that exclusion of lung tissue from the PTV during IMNI results in lower doses in the organs at risk. Despite daily imaging, it also leads to a significant reduction of the dose coverage in the internal mammary region and a large dose variability among the patients.

The ICRU defines the Planning Target Volume (PTV) as a “geometrical concept (…) to select appropriate beam size and beam arrangements taking into consideration the net effect of all possible geometrical variations and inaccuracies in order to ensure that the prescribed dose is actually absorbed in the CTV [25]”. Geometrical variations and inaccuracies during RNI are mostly related to patients positioning during radiotherapy and anatomical changes during the course of treatment. The suggested CTV to PTV margins for RNI in breast cancer vary between 5 mm and 10 mm [17,21,26,27]. However, the current literature lacks data and analyses regarding the PTV margins for modern treatment modalities such as VMAT or SIB irradiation. Feng et al. [28] investigated PTV margins in postmastectomy patients in 613 treatment fractions using kV data and found a margin of at least 4–8 mm must be retained despite the use of daily IGRT. Shah et al. [29] assessed the daily setup errors during 3DCRT using surface imaging. Averaged over all patients, the mean displacements were 4.1 ± 2.6 cm. Neither the ESTRO nor the RTOG contouring recommendations address PTV margins. The ESTRO explains this with the fact that PTV margins should be based on actual measurements of set-up. Still, not all clinics perform set-up measurements and evidence-based recommendations regarding the PTV margins could help to further improve the standardization and precision of radiotherapy.

The principle of excluding lung tissue for the PTV is widely used in clinical practice and has been applied in previous studies on adjuvant radiotherapy in breast cancer [17,20]. However, to our knowledge there is no evidence or scientific background regarding this practice. Assessment of PTV margins is difficult as the actual “delivered” dose to the CTV remains unclear during and after the treatment. Therefore, PTV margins are often evaluated by measurement of the maximal and median set-up errors [28]. Dose recalculation and accumulation based on daily CBCTs, as implemented in the current study, is a more accurate approach as it takes intra-fractional changes into account and allows comparison of the dose distribution in the clinical target volume in dependence of different PTV margins.

In our study, exclusion of overlapping lung tissue from the PTV during IMNI leads to significant dose reduction in the CTV-IMN during radiotherapy compared to conventional 5 mm PTV margins. This was the case although the patient position was adjusted daily by CBCT. This is attributable to the fact that CBCT cannot compensate all inaccuracies such as tissue swelling or breast position and the remaining variability demands sufficient safety margins. If IGRT is performed on a weekly basis or every other day, larger inter-fractional set up inaccuracies need to be expected. This would most certainly result in a further decrease of the dose coverage in the CTV-IMN if overlapping lung tissue is excluded from the PTV.

Dose prescription for IMNI ranges from 45 to 50.4 Gy in prospective studies [30]. However, the actual dose delivered to the IMN was probably lower in most patients: The quality assurance of “conventional non-CT-based internal mammary lymph node irradiation” in the DBCG-IMN study (prescribed dose to IMN: 48 Gy) revealed that IMN-V90% ranged in dependence of the technique between 73.4% and 86.9% [31]. Furthermore, 3D- plan simulations of the EORTC and MA.20 treatment fields, resulted in a mean dose of only 41.8 Gy and 37.8 Gy in the INM (for a standard patient, prescribed dose 50 Gy) [32]. Nevertheless, incomplete IMN coverage in the prospective trials does not justify reduction of PTV margins. During VMAT irradiation or wide tangents, the PTV margins are of particular importance as the anterior-posterior dose gradient is steeper compared to treatment with anterior parasternal fields (used in the EORTC and MA.20 trial). Excluding lung tissue from the PTV reduces the PTV margin to 0 mm in some parts of the target volume, which leads to accidental, uncontrollable dose reduction. This is illustrated by the wide range of V90% to V95% of the IMN-CTV among the patients using the Ex-Lung PTV in Figure 4. To ensure standardized and sufficient treatment for all patients, the use of a sufficient CTV-PTV margin is necessary. Instead of reducing the PTV margin, reduction of the prescribed to 45 Gy for the IMN can be discussed in patients with lower risk factors. For patients with IMN positive lymph nodes, however, there is emerging evidence that IMN irradiation with higher doses (IMNI ≥ 63.6 Gy or boost-RT to metastases) is associated with a better DFS compared to lower doses (50.0–63.5 Gy) and excellent IMN control [33]. This emphasizes the particular importance of adequate dose coverage and sufficient PTV-margins in these patients.

In addition to set-up accuracies, the PTV margins also accounts for inaccuracies of linear accelerators and treatment planning as well as intra-fractional movement. Reitz et al. [34] analyzed intra-fractional movement in 2028 breast cancer patients using a surface scanner. The maximum magnitude of the deviation vector showed a mean change of 1.93 mm ± 1.14 mm (standard deviation [SD]) (95% confidence interval: [0.48–4.65] mm). These uncertainties strengthen the elevated concerns regarding exclusion of overlapping lung tissue from the PTV.

There are several established techniques to achieve a better sparing of the organs at risk including deep inspiration breath hold, prone positioning and partial breast irradiation [35]. Compared to these techniques, exclusion of lung tissue from PTV had a comparably small effect on the OAR dose in our study. Due to the uncontrollable reduction of dose coverage in the target volume, it cannot be considered a valid technique for OAR sparing. Instead, (at least) a 5 mm PTV margin should be used around the IMN-CTV in all directions. The dose to the OARs in the current study was comparably high, since dose coverage in the target volume was prioritized over optimal OAR-sparing. Whether PTV dose coverage can be compromised in some cases in order to spare OAR dose was beyond the scope of the current investigation and should be addressed in further studies.

The additional toxicity of IMNI (using a 5 mm PTV margin) is low for most patients when DIBH is being used [14]. Therefore, DIBH should be considered as preferred technique to reduce the dose in the OARs instead of excluding the overlapping lung tissue.

## 5. Conclusions

Reduction of PTV margins around IMN by exclusion of lung tissue leads to accidental reduction of dose coverage in the IMN-CTV. Daily imaging cannot completely compensate for anatomic changes and intra-fraction movement. Therefore, exclusion of overlapping lung tissue from the PTV should not be performed as a standard procedure to reduce the dose in the OARs.

## Data Availability

The data presented in this study are available on request from the corresponding author. The data are not publicly available due to ethical reasons.
